# Chorioallantoic membranes of embryonated chicken eggs as an alternative system for isolation of equine influenza virus

**DOI:** 10.1186/s12985-017-0788-3

**Published:** 2017-06-21

**Authors:** Ilona Marcelina Gora, Malgorzata Kwasnik, Jan Franciszek Zmudzinski, Wojciech Rozek

**Affiliations:** Department of Virology, National Veterinary Research Institute, Partyzantow 57, 24 100 Pulawy, Poland

**Keywords:** Influenza virus, Virus isolation, Chorioallantoic membranes

## Abstract

**Background:**

Influenza virus isolation in embryonated chicken eggs (ECEs) is not applicable for rapid diagnosis, however it allows the recovery and propagation of the viable virus. A low number of infectious virus particles in the swabs, poor quality of samples or individual strain properties can lead to difficulties during the virus isolation process. We propose to utilize chorioallantoic membranes (CAM) of ECEs with the assistance of real-time RT PCR to facilitate equine influenza virus isolation.

**Methods:**

Real-time RT PCR was used to detect influenza virus genetic material in amniotic/allantoic fluids (AF) and CAM of ECEs. Haemagglutination assay was used for AF. We used highly diluted virus as a substitute of clinical specimen for ECEs inoculation.

**Results:**

Our study demonstrated that real-time RT PCR testing of CAM homogenates was more useful than testing of AF for EIV detection in ECEs. Positive results from CAM allowed to select the embryos from those with haemagglutination assay (HA) - and real-time RT PCR-negative AF for further passages. Using homogenates of CAM for subsequent passages, we finally obtained HA-positive AF, which confirmed virus replication.

**Conclusion:**

We postulate that real-time RT PCR testing of CAM homogenates and their subsequent passages may facilitate the isolation of equine influenza viruses.

**Electronic supplementary material:**

The online version of this article (doi:10.1186/s12985-017-0788-3) contains supplementary material, which is available to authorized users.

Equine influenza has been diagnosed both in vaccinated and unvaccinated horses, however the infection was not always confirmed by virus isolation [1]. Detection of equine influenza virus (EIV) directly from nasopharyngeal swabs of horses is usually performed using immunoenzymatic antigen detection methods (e.g. ELISA, rapid antigen detection tests) or viral nucleic acid amplification [2, 3]. Real-time RT PCR (rtRT PCR) is the most sensitive assay for EIV detection currently available [4, 5]. Virus isolation in embryonated chicken eggs (ECEs) or Madin-Darby canine kidney cells is not required for rapid diagnosis, however it allows the recovery and propagation of the viable viral strains, which is essential for virus characterization. Traditionally, ECEs have been preferred for isolation of EIV and some authors proved that ECEs were more effective for virus isolation than cell cultures [6]. According to the OIE Terrestrial Manual, amniotic/allantoic fluids (AF) are tested by haemagglutination assay (HA) and passaged into new eggs. If the HA titre is 16 or more, isolates are characterized immediately. If the HA titre is low or no haemagglutination activity is found, aliquots of AF are pooled and further passaged in ECEs. If virus is not recovered by the fifth passage, further passages are not likely to be successful [7]. To recover some influenza virus strains from clinical material, more sensitive assays for virus detection in ECEs are needed. Additionally to HA of AF, we examined chorioallantoic membranes (CAM) and AF by rtRT PCR for the detection of M gene of influenza A virus. CAM homogenates from ECEs inoculated with highly-diluted EIV were used for subsequent passages until virus was detectable in AF by HA.

EIV strain A/equine/Pulawy/2008 (H3N8) having titre 10^7.1^ EID50/ml (3rd passage in ECEs) was serially diluted from 10^−1^ to 10^−8^. Six 9-day-old ECEs were inoculated with 0.2 ml of each dilution into the allantoic sac. After 72 h incubation at 37 °C ECEs were cooled down to 4 °C. After AF have been collected, the body of embryo and yolk membrane were removed from the egg shell. CAM were separated out with sterile tweezers. Each CAM was rinsed in cold PBS, weighted, frozen in -70^o^ C and defrosted. To each CAM three volumes (*v*/*w*) of PBS were added and samples were sonicated (30 s, 130 W). Haemagglutination titre was determined for AF. RNA was extracted from AF and CAM homogenates using TRI Reagent® (Sigma-Aldrich, St. Louis, USA) as described by Chomczynski and Sacchi [8]. The TaqMan rtRT PCR was performed using a QuantiTect Probe RT-PCR kit (Qiagen, Hilden, Germany) on the 7500 Fast real-time PCR system (Applied Biosystems, Foster City, USA) according to the protocol recommended by WHO [9]. Primers and the probe used in this study targeted the conservative influenza virus M gene: InfA Forward 5′-GACCRATCCTGTCACCTCTGAC-3′; InfA Reverse 5′-AGGGCATTYTGGACAAAKCGTCTA-3′; InfA Probe1 5′-FAM TGC AGT CCT CGC TCA CTG GGC ACG –BHQ1–3′. The reaction was conducted in a total volume of 25 μl containing 0.4 μM of each primer, 0.12 μM of probe and 2 μl of viral RNA. Reaction conditions were as follows: one cycle at 45 °C for 10 min, followed by 10 min at 95 °C, and 40 cycles of 15 s at 95 °C and 45 s at 60 °C. Statistical analysis was done based on six replicates of both AF and CAM for each dilution. Mann - Whitney *U* test [10] was used to describe differences in C_t_ values between CAM and AF.

A haemagglutination assay was used for AF from ECEs inoculated with 10^−1^ to 10^−8^ . HA titers 64 and above were obtained for the dilution range 10^−1^ to 10^−5^. For 10^−6^ dilution 50% of ECEs were HA positive and the highest standard deviation (SD) of Ct was observed (Table 1). Using higher viral dilutions we obtained ECEs with no HA titres. For this group we tested AF and CAM with rtRT PCR. The mean C_t_ values were lower for CAM than for AF in dilutions 10^−5^ to 10^−8^. Positive results in rtRT PCR (C_t_ < 38) were obtained for ECEs inoculated by 10^−6^ EIV dilution for AF and by 10^−8^ (mean C_t_ = 33.15) for CAM homogenates. The Ct 38 threshold for positive results was based on O.I.E. protocol recommendations [9]. AF from infected ECEs (HA 256) and AF from uninfected ECEs (HA negative) were used as positive and negative controls, respectively. Ct values in the range from 14.1 to 15 for positive and >38.9 for negative controls were obtained. Beta actin gene was used as internal control of rt RT PCR and mean Ct value 30.94 and 19.15 were obtained for AF and CAM, respectively. There was no correlation between Ct values for M and beta actin genes. Results are presented in supplementary materials (“Additional file 1”). Our study showed that rtRT PCR testing of CAM homogenates was more helpful than rtRT PCR of AF for EIV detection in ECEs. Inoculation of low doses of virus (10^−7^ and 10^−8^ dilutions) resulted in negative HA titre as well as negative rtRT PCR of AF, while results of rtRT PCR for CAM showed the presence of the viral genetic material. Detection of EIV genetic material in ECEs is not the proof of the presence of viable virus, however, it may indicate the potential virus source.

**Table 1:** Real-time RT PCR of amniotic/allantoic fluids (AF) and chorioallantoic membranes (CAM) from embryonated chicken eggs inoculated with A/equine/Pulawy/2008

Dilution	10^−5^		10^−6^		10^−7^		10^−8^	
Sample	AF	CAM	AF	CAM	AF	CAM	AF	CAM
Ct (*n* = 6)	19.3^a^	11.3	36	27.4	40	32.1	40	29.7
	19.3^b^	12.3	18.4^a^	8.5	40	32	40	34.6
	19.4^a^	12.2	19^a^	13.3	40	32.7	40	33.1
	14.5^b^	12.6	35.6	24.9	40	30.1	40	34.9
	16.3^b^	12.4	18.2^a^	8.9	40	33.9	38.9	34.2
	19.5^a^	11.5	35.9	24.3	40	30.8	40	32.4
Mean	18.05	12.05	27.18	17.88	40	31.93	39.81	33.15
SD	2.1	0.5	9.5	8.6	0	1.4	0.4	1.9
p		0.0050		0.1735		0.0028		0.0037

^a^HA 64

^b^HA 128

*SD* standard deviation

*p* - *p* value calculated with Mann–Whitney *U* test

In the second part of the experiment, passages of pooled CAM homogenates and AF harvested from ECEs inoculated with the virus A/equine/Pulawy/2008 diluted from 10^−6^ to 10^−8^ were performed. The first passage was done with ECEs, which AF were previously confirmed negative in HA. Inoculation with the highest dilution (10^−8^) was triplicated. We tested if subsequent passages of CAM homogenates would result in HA-positive AF. Positive HA results for AF were obtained in the first passage of CAM homogenates derived from ECEs inoculated with 10^−6^ virus dilution. For more diluted inoculum, positive HA titres of AF were obtained in the second or third passages of 10^−7^ and 10^−8^ dilutions, respectively (Table 2). Passages of AF resulted in negative HA (HA < 4) of AF by the third passage (except for one sample with HA 8 in the third passage of AF derived from embryo inoculated with 10^−7^ dilution). AF samples were tested also by rtRT PCR for M gene and Ct values below 38 were obtained in all cases (data included in “Additional file 2”).

**Table 2:** HA titres of amniotic/allantoic fluids (AF) in three consecutive passages of AF or chorioallantoic membranes (CAM) homogenates

CAM						AF				
Dilution	10^−6^	10^−7^	10^−8^	10^−8^	10^−8^	10^−6^	10^−7^	10^−8^	10^−8^	10^−8^
1st passage	128	<4	<4	<4	<4	<4	<4	<4	<4	<4
(*n* = 3)	128	<4	<4	<4	<4	<4	<4	<4	<4	<4
	256	<4	<4	<4	<4	<4	<4	<4	<4	<4
2nd passage		128	<4	<4	<4	<4	<4	<4	<4	<4
(*n* = 5)		<4	<4	<4	<4	<4	<4	<4	<4	<4
		256	<4	<4	<4	<4	<4	<4	<4	<4
		128	<4	<4	<4	<4	<4	<4	<4	<4
		128	<4	<4	<4	<4	<4	<4	<4	<4
3rd passage			128	32	256	<4	<4	<4	<4	<4
(*n* = 5)			64	32	64	<4	<4	<4	<4	<4
			128	64	1024	<4	<4	<4	<4	<4
			128	8	2048	<4	8	<4	<4	<4
			64	64	128	<4	<4	<4	<4	<4

A low number of infectious viral particles in the swabs, poor quality of samples or individual strain properties can lead to difficulties in the influenza virus isolation. The possibility of ECEs preselection for pooling samples could eventually increase the isolation rate if the virus is present in samples at a very low concentration. In this study we used rtRT PCR detection of EIV in combination with consecutive passages of CAM homogenates. Influenza virus could infect the layer of susceptible cells covering CAM. Chorioallantoic membranes function as the respiratory organs of the embryo and as reported previously are probably the most abundant source of virus in eggs [11]. Our rtRT PCR test results showed a higher level of EIV genetic material in CAM than in AF. Influenza virus is transmitted from cell to cell in cell-free form, after budding from the cell surface, which is facilitated by neuraminidase activity. Progeny virions might also pass on to adjacent, uninfected cells when they remain associated with the parental cell surface [12]. Another mode of cell to cell transmission is direct translocation of viral ribonucleoprotein complexes via intercellular connections and infection of neighboring cells [13]. This might explain, why genetic material of influenza virus is detected in CAM while AF from the same ECE is negative. Other components of ECEs than AF were used by Tang et al. [14] in influenza virus isolation. The authors showed that passaging of mixed chick embryo and AF improved the isolation rate of avian influenza virus from field samples under non-optimal conditions (old fecal samples). CAM fragments maintained in roller tubes were used by Samuel et al. in 1981 for respiratory virus isolation and multiplication [15]. They used haemagglutination assay for confirmation of influenza A and B virus multiplication in in vitro CAM cultures inoculated with human clinical material. We used rtRT PCR as a highly sensitive detection method to confirm EIV multiplication in CAM.

In summary, we suppose that the monitoring of CAM homogenates from inoculated ECEs by rtRT PCR and their subsequent passages could possibly facilitate the isolation of EIV. Further experiments using more strains and clinical material are needed. The presented approach was used for equine influenza virus however, it probably could be used for influenza A viruses from other hosts.

## Additional files

Additional file 1: Real time RT PCR for beta actin gene in AF and CAM of embryos inoculated with A/equine/Pulawy/2008. (XLSX 35 kb)

Additional file 2: Real time RT PCR for M and beta actin genes in three consecutive passages of AF and CAM. (XLSX 183 kb)

## Abbreviations

AF: Amniotic/allantoic fluids
CAM: Chorioallantoic membranes
Ct: Cycle threshold
ECEs: Embryonated chicken eggs
EID50: 50% Egg Infective Dose
EIV: Equine influenza virus
HA: Haemagglutination assay
OIE: World Organisation for Animal Health
rtRT PCR: Real-time Reverse Transcription Polymerase Chain Reaction
SD: Standard Deviation

## References

[1] Slater J, Borchers K, Chambers T, Cullinane A, Duggan V, Elton D, Legrand L, Paillot R, Fortier G (2014). Report of the international equine influenza roundtable expert meeting at le Touquet, Normandy, February 2013. Equine Vet J.

[2] Galvin P, Gildea S, Nelly M, Quinlivan M, Arkins S, Walsh C, Cullinane A (2014). The evaluation of three diagnostic tests for the detection of equine influenza nucleoprotein in nasal swabs. Influenza Other Respir Viruses.

[3] Oxburgh L, Hagström A (1999). A PCR based method for the identification of equine influenza virus from clinical samples. Vet Microbiol.

[4] Lu Z, Chambers TM, Boliar S, Branscum AJ, Sturgill TL, Timoney PJ, Reedy SE, Tudor LR, Dubovi EJ, Vickers ML (2009). Development and evaluation of one-step TaqMan real-time reverse transcription-PCR assays targeting nucleoprotein, matrix, and hemagglutinin genes of equine influenza virus. J Clin Microbiol.

[5] Quinlivan M, Dempsey E, Ryan F, Arkins S, Cullinane A (2005). Real-time reverse transcription PCR for detection and quantitative analysis of equine influenza virus. J Clin Microbiol.

[6] Quinlivan M, Cullinane A, Nelly M, Van Maanen K, Heldens J, Arkins S (2004). Comparison of sensitivities of virus isolation, antigen detection, and nucleic acid amplification for detection of equine influenza virus. J Clin Microbiol.

[7] OIE (Office Internationale des Epizooties) 2015. Equine Influenza. In: Manual for Diagnostic Tests and Vaccines for Terrestrial Animals. Accessed Paris 2015: http://www.oie.int/fileadmin/Home/eng/Health_standards/tahm/2.05.07_EQ_INF.pdf

[8] Chomczynski P, Sacchi N (1987). Single-step method of RNA isolation by acid guanidinium thiocyanate-phenol-chloroform extraction. Anal Biochem.

[9] World Health Organization (WHO), 2013. Real-time RT PCR Protocol for the Detection of Avian Influenza A (H7N9) Virus. Accessed 8 Apr 2013: http://www.who.int/influenza/gisrs_laboratory/cnic_realtime_rt_pcr_protocol_a_h7n9.pdf

[10] Mann HB, Whitney DR (1947). On a test of whether one of two random variables is stochastically larger than the other. Ann Math Statistics.

[11] Hardy CT, Young SA, Webster RG, Naeve CW, Owens RJ (1995). Egg fluids and cells of the chorioallantoic membrane of embryonated chicken eggs can select different variants of influenza a (H3N2) viruses. Virology.

[12] Mori K, Haruyama T, Nagata K (2011). Tamiflu-resistant but HA-mediated cell-to-cell transmission through apical membranes of cell-associated influenza viruses. PLoS One.

[13] Roberts KL, Manicassamy B, Lamb RA (2015). Influenza a virus uses intercellular connections to spread to neighboring cells. J Virol.

[14] Tang S, Li Y, Xia H, Huang J, Zhang Z, Zhu N, Zhao J, Li T (2014). Improved methods for isolation of avian influenza virus. J Virol Methods.

[15] Samuel I, Mihail A, Teodosiu O, Barnaure F, Coban E, Petrescu A, Matinca D (1981). Comparative results of respiratory virus isolation attempts in chorioallantoic membrane fragments and embryonated chicken eggs. Virologie.

